# Cholera Outbreaks in Low- and Middle-Income Countries in the Last Decade: A Systematic Review and Meta-Analysis

**DOI:** 10.3390/microorganisms12122504

**Published:** 2024-12-04

**Authors:** Anastasia A. Asantewaa, Alex Odoom, Godfred Owusu-Okyere, Eric S. Donkor

**Affiliations:** 1 Department of Medical Microbiology, University of Ghana Medical School, Korle Bu, Accra P.O. Box KB 4236, Ghana; asantewaaaa11@gmail.com (A.A.A.); alexodoom2018@gmail.com (A.O.); 2 National Public Health & Reference Laboratory (NPHRL), Ghana Health Service-Korle Bu, Accra P.O. Box 300, Ghana; godow2000@yahoo.com

**Keywords:** cholera, *Vibrio cholerae*, outbreaks, low- and middle-income countries (LMICs), developing countries

## Abstract

Cholera is linked to penury, making low- and middle-income countries (LMICs) particularly vulnerable to outbreaks. In this systematic review, we analyzed the drivers contributing to these outbreaks, focusing on the epidemiology of cholera in LMICs. This review followed the Preferred Reporting Items for Systematic Reviews and Meta-Analyses (PRISMA) guidelines and was registered in PROSPERO (ID: CRD42024591613). We searched PubMed, Scopus, Web of Science, and Google Scholar to include studies on cholera outbreaks that occurred in LMICs from 1 January 2014 to 21 September 2024. Studies on outbreaks outside LMICs and focusing on sporadic cases were excluded. The risk of bias among included studies was assessed using a modified Downes et al. appraisal tool. Thematic analysis was used to synthesize the qualitative data, and meta-analyses to estimate the pooled prevalence. From 1662 records, 95 studies met inclusion criteria, primarily documenting outbreaks in Africa (74%) and Asia (26%). Contaminated water was the main route of disease transmission. The pooled fatality prevalence was 1.3% (95% CI: 1.1–1.6), and the detection rate among suspected cases was 57.8% (95% CI: 49.2–66.4). *Vibrio cholerae* O1 was the dominant serogroup while Ogawa was the dominant serotype. All studies reporting biotypes indicated El Tor. Although the isolates were 100% susceptible to ofloxacin, levofloxacin, norfloxacin, cefuroxime, and doxycycline, they were also fully resistant to amikacin, sulfamethoxazole, trimethoprim, and furazolidone. The persistence of cholera outbreaks in destitute areas with limited access to clean water and sanitation emphasizes the need for socioeconomic improvements, infrastructure development, and ongoing surveillance to support timely responses and achieve long-term prevention.

## 1. Introduction

Cholera is an acute diarrheal disease caused by the toxigenic strains of *Vibrio cholerae*, particularly serogroups O1 and O139 [1]. With a short incubation period of two hours to five days, the disease can spread quickly through fecal contamination of water or food, resulting in explosive outbreaks that can overwhelm local health infrastructure [1]. While cholera can be asymptomatic or mild in some cases, severe infections can progress rapidly, with patients losing large volumes of fluid in a short period, making timely treatment critical to reducing mortality [2].

The recurrence of cholera is largely due to poor infrastructure and poverty, making LMICs especially vulnerable to repeated outbreaks [3]. Historically, the disease has been endemic in the Asian subcontinent and has triggered seven pandemics since 1817, with the most recent beginning in 1961 and still ongoing [4]. In recent years, cholera outbreaks have been reported in multiple countries. In 2021, 23 countries, mostly in the World Health Organisation (WHO) regions of Africa and the Eastern Mediterranean, experienced outbreaks. By November 2022, cholera cases had been documented in over 29 countries, with 16 nations suffering from prolonged outbreaks [2]. Yemen, for instance, saw over 2.5 million suspected cases and 4000 deaths between 2016 and 2022, marking one of the worst cholera outbreaks in modern history [5].

Although global efforts have been made to control cholera, the actual impact of the disease is likely much higher than reported figures suggest. The WHO estimates that cholera causes 3 to 5 million cases and 100,000 to 120,000 deaths each year, but many cases go unreported due to weak surveillance systems, resource constraints, and the stigma associated with the disease [6]. Additionally, cholera is often difficult to distinguish from other diarrheal diseases based solely on symptoms, complicating outbreak detection and management. 

Many studies have documented cholera outbreaks in developing nations. However, a systematic examination of their causes and dynamics is lacking. Recent reviews [3,7] mainly focused on specific regions, such as India and Sub-Saharan Africa. To bridge this gap, we conducted a broader analysis of cholera outbreak characteristics and transmission dynamics across various LMICs. The results aim to provide a clear overview of cholera outbreaks and to act as a valuable resource for public health officials in controlling and managing the disease.

## 2. Materials and Methods

### 2.1. Search Strategy

This systematic review followed the PRISMA guidelines (Supplementary Table S1), [8] and was registered in PROSPERO (ID: CRD42024591613). We searched electronic databases including MEDLINE through PubMed, Scopus, Web of Science, and Google Scholar. The search covered studies published in English between 1 January 2014 and 21 September 2024. Keywords such as “cholera”, “outbreak”, and “cholera infection” were combined with specific names of LMICs to focus on relevant countries. The detailed search strategy is provided in Supplementary Table S2.

Search results were exported to Rayyan [9], where duplicates were identified and manually removed. Two reviewers screened the remaining studies by title and abstract, followed by full-text reviews to confirm eligibility. Any disparities regarding final study inclusion between the two reviewers were recognized and resolved through discussion and consensus. A third reviewer was available to mediate if consensus was not achieved; however, no intervention was needed.

### 2.2. Eligibility Criteria 

#### 2.2.1. Inclusion Criteria

Studies met the inclusion criteria if they:

- Reported epidemiological data, including transmission routes and risk factors from a confirmed cholera outbreak in countries classified as LMICs by the World Bank [10];
- Reported cholera outbreaks that occurred between 1 January 2014 and 21 September 2024;
- Were original research articles with a focus on outbreak epidemiology. Study designs could be cross-sectional, cohort, case–control, or surveillance-based outbreak reports.

#### 2.2.2. Exclusion Criteria

Studies were excluded if they:

- Focused only on sporadic, endemic cholera cases rather than outbreak settings;
- Only presented data as conference abstracts without full-text availability;
- Were reviews, editorials, commentaries, and discussion pieces without primary epidemiological findings;
- Presented epidemiological models or simulations rather than empirical outbreak investigation results;
- Pertained to high-income countries, or cholera-endemic regions rather than outbreak events;
- Addressed interventions, candidate vaccines, or treatment effectiveness rather than descriptive epidemiology.

### 2.3. Quality Assessment

The risk of bias in the included studies was evaluated by two reviewers using a modified Downes et al. appraisal checklist for cross-sectional studies (AXIS) [11]. The key factors evaluated were the clarity of the study objectives, description of the study design, and the presence of a clear definition of cholera. The appraisal tool also examined sampling methods, reporting of outcomes such as risk factors and case fatality rates, and the rigor of data collection and statistical analysis. Additionally, the use of diagnostic methods such as culture or PCR for cholera confirmation, along with discussions of limitations and confounders, was considered. Based on these criteria, each study was rated as having a low, moderate, or high risk of bias. 

### 2.4. Data Extraction and Synthesis

Data, including geographic location, region, study design, and timeframe of the cholera outbreak, were extracted using an Excel spreadsheet (Version 2410). Variables like the number of cases, transmission routes, and case fatality ratios (CFR) were also captured, as well as any identified risk factors such as water contamination and poor sanitation. Additionally, details on the *V. cholerae* serogroup, serotypes, and biotypes were documented, and antibiotic resistance data were included where applicable. The results were thematically synthesized and presented in textual narrative formats, tables, and figures. A random-effects meta-analysis with 95% confidence intervals was conducted to estimate the pooled prevalence of laboratory-confirmed cases and overall disease fatality. Heterogeneity was statistically assessed using the I^2^ metric and Chi-squared test. Subgroup analyses explored potential sources of heterogeneity by region. Publication bias was statistically assessed using Egger’s tests, and graphically using funnel plots.

## 3. Results

### 3.1. Search Results

**Figure 1:**
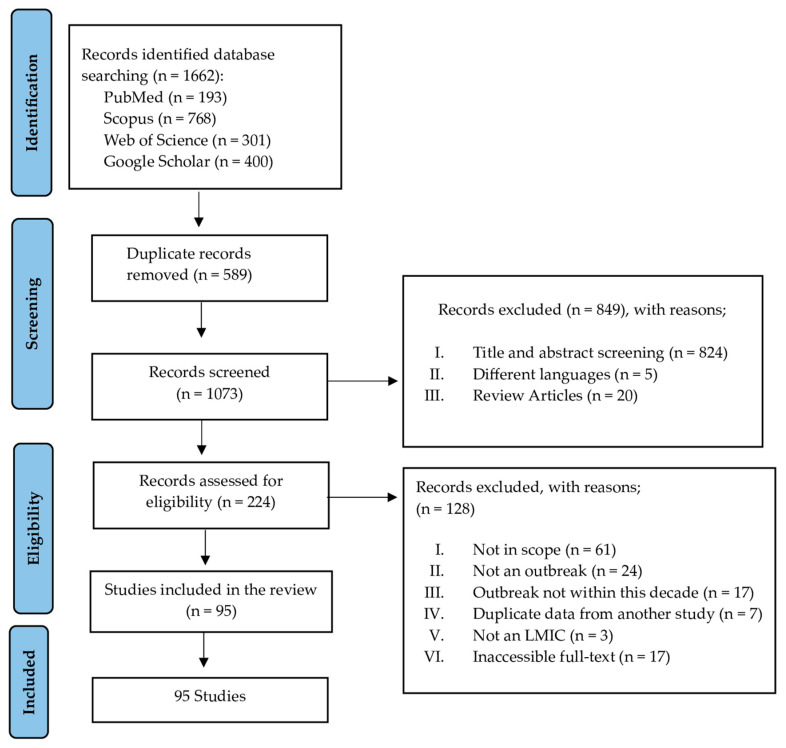
PRISMA flowchart for study selection.

In total, 1662 records were identified through searches in PubMed (*n* = 193), Scopus (*n* = 768), Web of Science (*n* = 301), and the first 400 results from Google Scholar. After removing 589 duplicates, 1073 records were screened. Of these, 847 were excluded due to irrelevance based on the title and abstract, language limitations, and review articles. This left 224 records for eligibility assessment, from which 128 were excluded for reasons such as not fitting the scope, not being outbreak studies, occurring outside the decade under review, non-LMIC settings, being duplicates, or inaccessible full texts. Ultimately, 95 studies were included in the final review [12,13,14,15,16,17,18,19,20,21,22,23,24,25,26,27,28,29,30,31,32,33,34,35,36,37,38,39,40,41,42,43,44,45,46,47,48,49,50,51,52,53,54,55,56,57,58,59,60,61,62,63,64,65,66,67,68,69,70,71,72,73,74,75,76,77,78,79,80,81,82,83,84,85,86,87,88,89,90,91,92,93,94,95,96,97,98,99,100,101,102,103,104,105,106] (Figure 1).

### 3.2. Risk of Bias

Of the 95 studies assessed, 44 (46.3%) were classified as having a low risk of bias, 41 (43.2%) were rated as moderate risk, and 10 (10.5%) were scored as high risk (Table S3).

### 3.3. Study Characteristics

**Figure 2:**
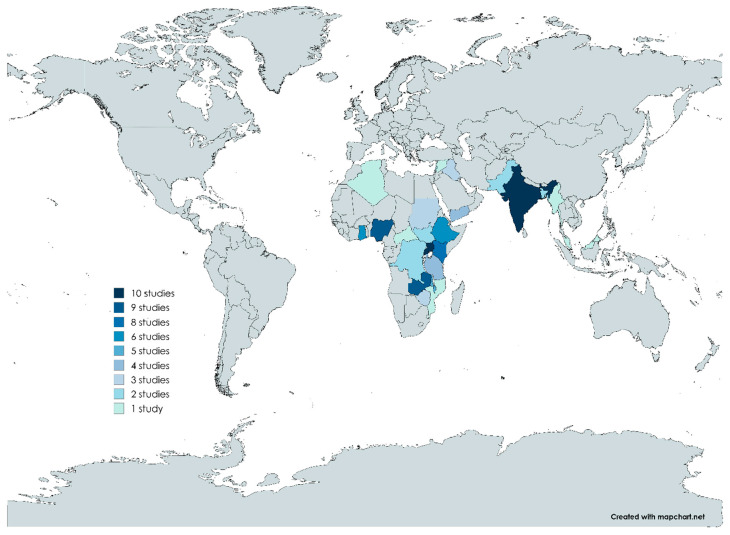
Distribution of studies across various LMICs. Created with MapChart, https://www.mapchart.net/world-advanced.html, accessed on 9 October 2024.

The 95 included studies reporting on cholera outbreaks across 24 countries were all recognized as LMICs by the World Bank [10]. The studies were primarily from Africa (*n* = 70; 74.0%) and Asia (*n* = 25; 26.0%), with most of them from India and Uganda, each with 10 studies (Figure 2). 

**Figure 3:**
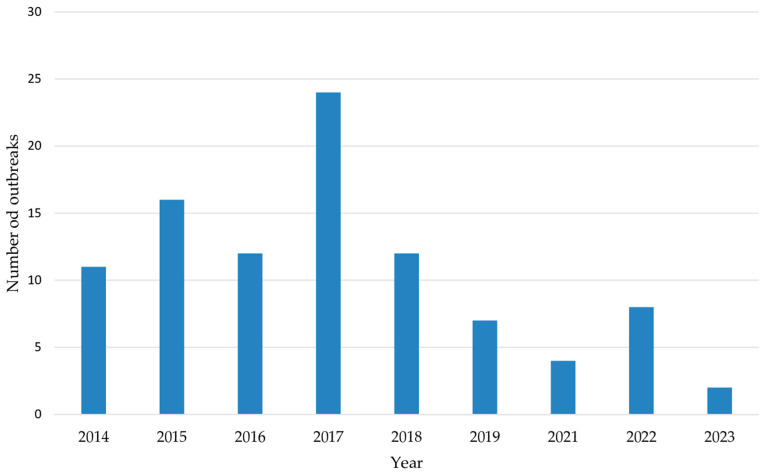
Number of cholera outbreaks by years of onset.

Cholera outbreaks documented in the included studies spanned from 2014 to 2024. While some studies reported protracted outbreaks covering multiple years, none reported an outbreak in 2020 (Figure 3, Table A1). The highest number of outbreaks began in 2017 (Figure 3), with several outbreaks continuing into subsequent years. The peak number of suspected cholera cases was recorded between 2016 and 2018, totaling over 2.3 million cases (Table A1). 

**Figure 4:**
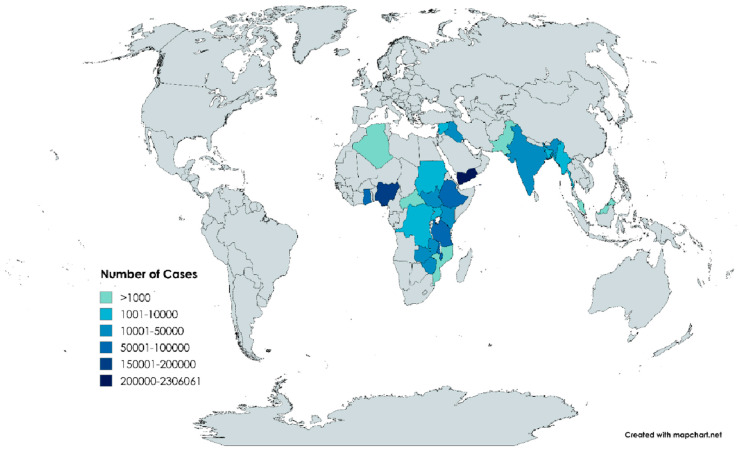
Number of cholera cases per country. Created with MapChart, https://www.mapchart.net/world-advanced.html, accessed on 9 October 2024.

More than 2.8 million suspected cases of cholera were reported. Yemen accounted for 80% of these cases, with 2.3 million suspected cases, whereas Malaysia reported the fewest cases, with only 78 cases. Other countries with substantial case numbers included Ghana, Nigeria, Malawi, and Tanzania, each reporting over 50,000 cases. Meanwhile, Pakistan, Algeria, the Central African Republic, and Mozambique reported fewer than 1000 cases (Figure 4, Table A1).

### 3.4. Routes of Cholera Transmission

**Figure 5:**
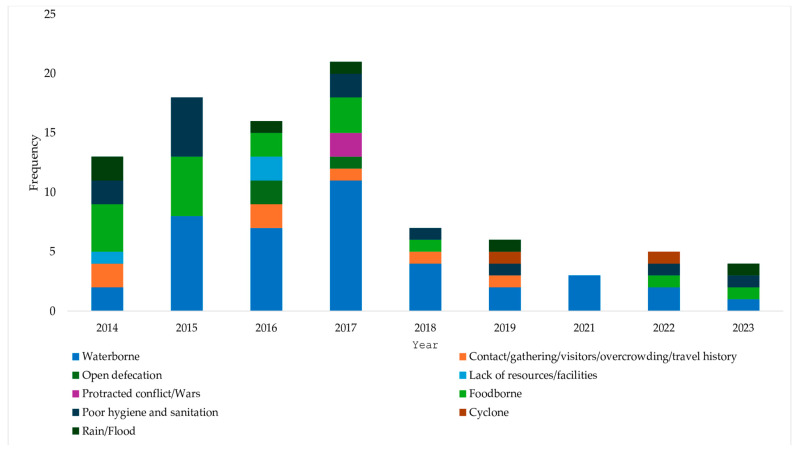
Transmission routes of cholera outbreaks by years of onset.

**Figure 6:**
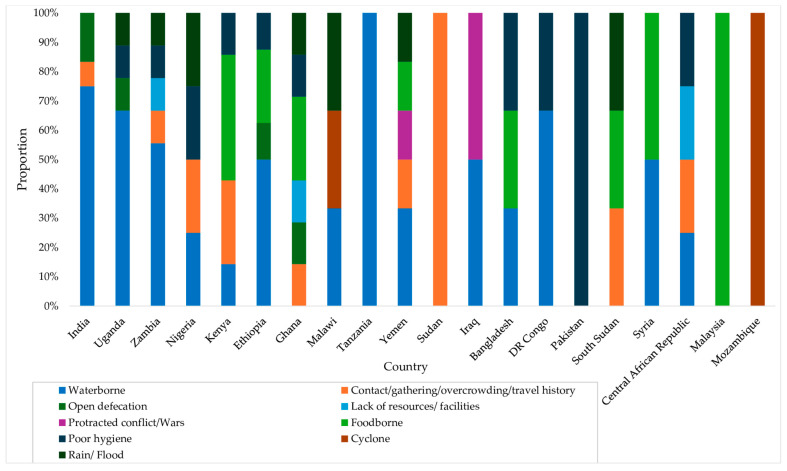
Transmission routes of cholera outbreaks by country.

Water contamination was the primary source of disease transmission throughout the decade (Figure 5) and across multiple countries (Figure 6). All outbreaks that began in 2021 were attributed to contaminated water sources. Moreover, it was reported as the sole transmission route for cholera outbreaks reported in Tanzania. Overcrowding and person-to-person contact were also noted as significant drivers of outbreaks in Kenya and Sudan, while open defecation played a role in Ghana, India, Uganda, and Ethiopia. Humanitarian crises and natural disasters such as protracted wars, floods, and cyclones also triggered outbreaks in Yemen, Iraq, Nigeria, Mozambique, Uganda, and Zambia, creating ideal conditions for cholera to spread across these regions. The other drivers were food contamination, poor hygiene and sanitation, and a lack of resources.

### 3.5. Pooled Prevalence of Cholera-Induced Fatality

**Figure 7:**
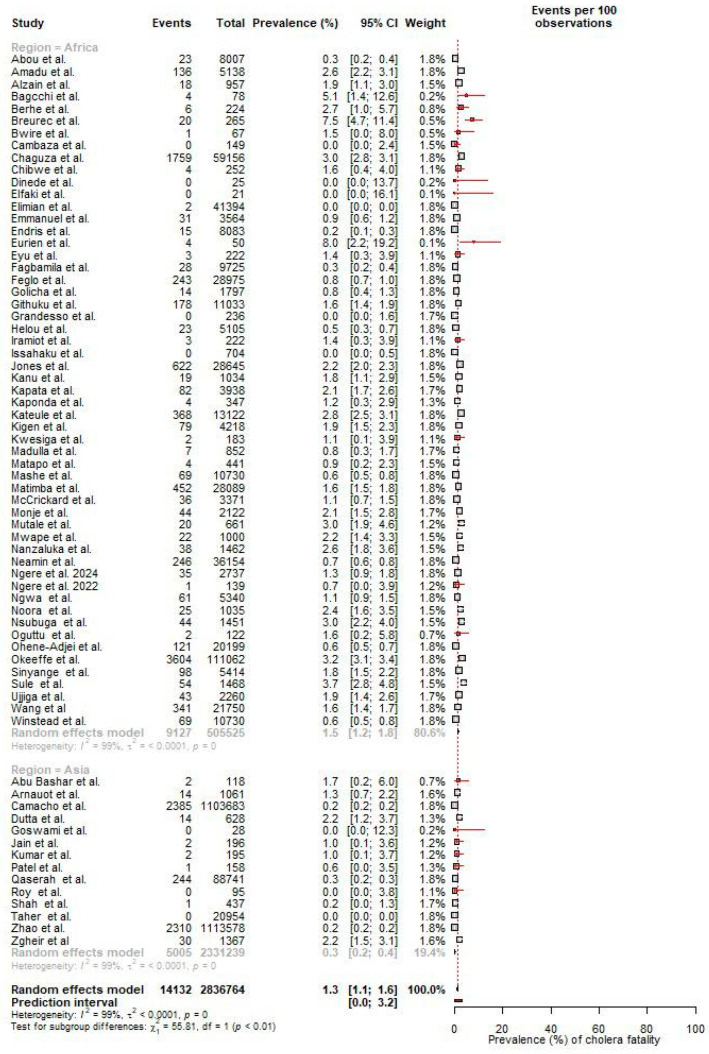
Pooled prevalence of fatalities during cholera outbreaks [12,13,14,15,16,18,20,23,24,25,26,27,29,34,36,38,39,40,41,42,43,44,49,50,51,52,53,54,55,56,58,60,61,62,63,65,66,67,68,69,70,71,73,74,76,78,80,81,82,83,84,85,86,87,88,89,92,93,95,97,98,99,100,101,102,103,104,105,106].

Based on a random-effect meta-analysis of 69 studies, the pooled fatality prevalence during outbreaks was 1.3% (95% Cl: 1.1–1.6). In terms of regional variation, Africa had a higher fatality prevalence (1.5%) compared to Asia (0.3%) (Figure 7). This variance suggests differing levels of outbreak impact or healthcare response effectiveness between the regions.

### 3.6. Laboratory-Confirmed Cholera Cases During Outbreaks

**Figure 8:**
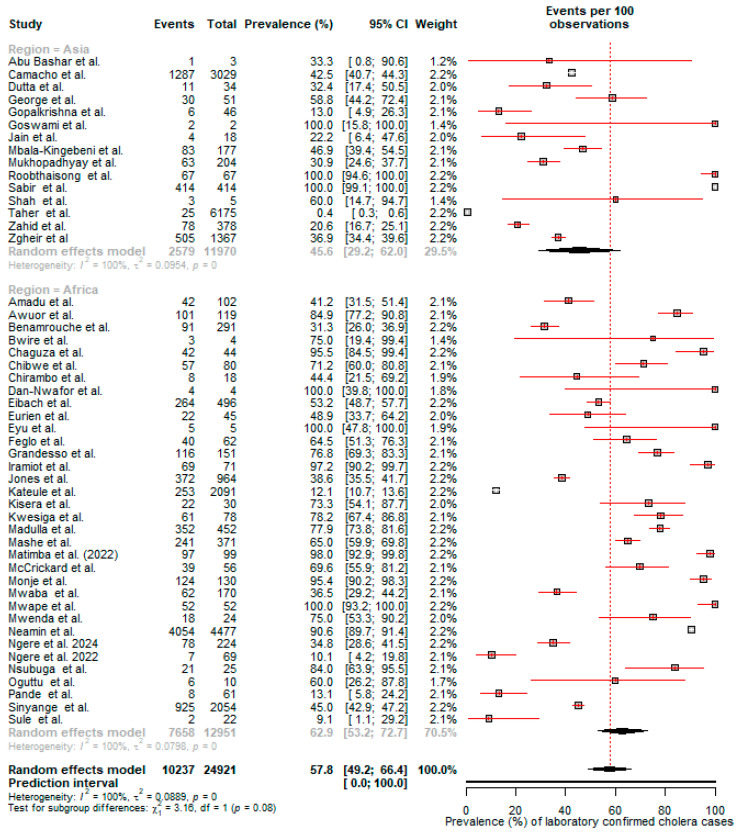
Pooled prevalence of laboratory-confirmed cholera during outbreaks [13,14,17,19,24,25,27,29,30,31,36,37,42,43,46,47,48,49,52,54,56,58,63,64,67,68,70,71,72,73,74,75,77,78,79,81,82,83,86,87,91,94,96,97,98,99,100,104,105].

The pooled detection rate of laboratory-confirmed cholera among suspected cases was 57.8% (95% Cl: 49.2–66.4) based on a random-effects meta-analysis of 49 studies. Africa had a higher rate of laboratory-confirmed cholera 62.9% compared to Asia (45.6%) (Figure 8).

### 3.7. V. cholerae Strain Classification

*V. cholerae* O1 was the predominant serogroup, accounting for 94.5% of the outbreaks, followed by *V. cholerae* O139 at 3.64% and non-O1/O139 at 1.81%. All identified isolates were classified under the El Tor biotype, with no instances of the classical biotype. Regarding serotypes, Ogawa was the most common (76.1%), Inaba comprised 15.2%, and a combination of both Ogawa and Inaba appeared in 0.065% of the reported outbreaks (Table A2).

### 3.8. Antimicrobial Resistance Patterns

**Figure 9:**
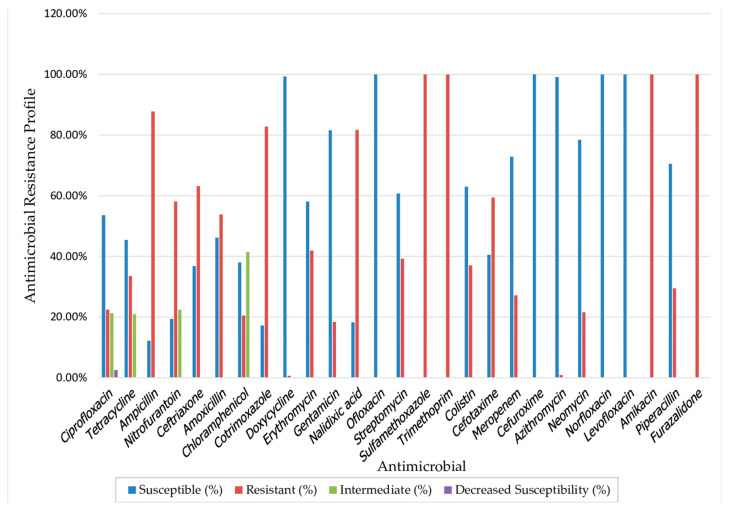
Antimicrobial resistance profiles of *V. cholerae* isolates (*n* = 799).

Data from 14 of 95 studies provided antimicrobial susceptibility testing (AST) results for 799 *V. cholerae* isolates against a range of antibiotics. Notably, several antibiotics exhibited high efficacy, with ofloxacin, levofloxacin, norfloxacin, cefuroxime, and doxycycline having susceptibility rates of at or near 100%. Conversely, ampicillin, cotrimoxazole, sulfamethoxazole, trimethoprim, amikacin, and furazolidone displayed extremely high resistance rates, with over 80% of isolates resistant, indicating their limited utility in treating these cholera strains (Figure 9).

## 4. Discussion

Since the seventh pandemic emerged in South Asia in 1961, cholera has not only continuously affected the region but also become endemic in several countries across Africa, Asia, and South America. In this review, while our focus was on low- and middle-income countries globally, we observed that the reported outbreaks in the included studies were in Africa (74.0%) and Asia (26.0%). Notably, Yemen recorded the highest number of suspected cases, with over 2.3 million cases reported during a protracted outbreak from 2016 to 2022 [25,35,93,106]. This outbreak, considered the worst cholera epidemic of modern times [107], was largely fuelled by the country’s ongoing civil war between pro-government forces and the Houthi armed movement, which severely weakened already fragile sanitation and healthcare systems [108].

Similarly, in Nigeria and the Democratic Republic of the Congo, conflict has been associated with increased rates of cholera outbreaks by 3.6 and 2.6 times, respectively [109]. Poor urban settlements, overcrowded conditions, and lack of access to water, sanitation, and hygiene make these areas particularly vulnerable to outbreaks during times of conflict [110]. In Syria, the destruction of water treatment facilities during an ongoing conflict left nearly half of the population relying on unsafe water sources to complement their daily water needs [16,111].

Other humanitarian crises caused by natural disasters such as floods, heavy rainfall, and cyclones were also identified as drivers of cholera outbreaks in this review [18,24,27,33,63]. For example, Cyclone Kenneth, which struck northern Mozambique in April 2019, severely damaged water and sanitation infrastructure, leading to a cholera outbreak [26]. Similarly, in Malawi, Cyclone Ana (January 2022) and Cyclone Gombe (March 2022) caused torrential rains and flooding, which overwhelmed water systems and overcrowded settlements, creating ideal conditions for cholera transmission [18,112]. Moreover, torrential rains and floods in early 2022 displaced many people in southern Malawi and left them without access to safe drinking water, further increasing the risk of cholera [18].

Across many cholera-prone areas, water contamination was identified as the primary transmission route, particularly in rural areas where rivers, hand-dug wells, deep wells, and springs serve as water sources [21,43,80,87,95]. These water sources are often poorly protected, making them unsafe for household use. Eyu et al. observed that community members used the river as a source of drinking water, as well as for washing clothes, kitchen utensils, and bathing, despite the river’s turbid and unprotected condition [43]. These unimproved water sources increase the odds of cholera infection by threefold [113]. Similarly, a significant link was found between drinking water from the Zamani River, a source of drinking water, and a cholera outbreak in a rural community in Nigeria [31]. In Ghana, Issahaku et al. found that pipelines passing through open drainage systems are highly susceptible to contamination, especially when damaged [55]. Furthermore, investigations during outbreaks have frequently revealed water sources contaminated with fecal matter, a major driver of *Vibrio cholerae* infections [55]. For instance, in North Karnataka, India, water samples were found to be heavily contaminated with fecal coliforms, indicating poor sewage disposal and unclean water sources [114]. Additionally, residents in Sembule Village, Uganda, were found to empty fecal waste into a drainage channel connected to a well (used as a drinking water source), particularly during rainfall when the waste was more likely to be washed away [42]. In another study in India, fecal matter was discovered in pond water that locals used for drinking and preparing fermented rice (Panta Bhat) [115]. Strikingly, the villagers believed that the fermented rice tasted better when prepared with pond water [115]. This indicates a lack of awareness of the health risks associated with contaminated water.

In countries such as India, Ghana, and Uganda, where open defecation is a persistent issue, it has been identified as a significant driver of cholera outbreaks [36,54,88,90]. Rainwater runoff frequently carries human waste into nearby rivers and water sources, worsening contamination and increasing the risk of *V. cholerae* infection [90]. According to studies by Iramiot et al. and Eyu et al., most latrines in affected areas are unimproved, which likely encourages widespread open defecation [43,54]. In Uganda, the National Housing and Population Census reported that while only 10% of the rural population lacks access to pit latrines, approximately 58% of the existing pit latrines are unimproved, contributing to the ongoing cholera risk [54].

While Challa et al. found that individuals who practiced water purification methods had a lower chance of contracting cholera [28], Davis et al. observed that despite extensive water chlorination efforts, cholera outbreaks were prolonged by the continued consumption of contaminated food in Ethiopia [32]. In many settings in Kenya, Malaysia, Ethiopia, Sudan, Bangladesh, and Syria, food contamination is a major cause of cholera outbreaks [16,41,47,50,57,79,88].

Research has consistently shown that certain foods, such as seafood, uncooked vegetables, and cold leftover rice, are vehicles for cholera outbreaks, as they may harbor *V. cholerae* strains [32,116]. These bacteria can thrive in improperly handled or stored food, making them a significant source of infection [32]. However, previous studies have indicated that heating food thoroughly can effectively kill *V. cholerae*, greatly reducing the risk of transmission [32,117]. In Jijiga, Ethiopia, a spike in cholera cases coincided with Ramadan, a time when there was an increase in street food vendors selling Iftar meals [32]. Anecdotal evidence suggests that many of these vendors were unlicensed and may not have followed proper food safety protocols, contributing to the outbreak. Similarly, in Ghana, where unlicensed street food vendors are prevalent [55], it was found that eating street-vended food was associated with a sixfold increase in the odds of contracting cholera [118].

Additionally, studies have consistently shown that attending social events where food is served, particularly in informal or outdoor settings, significantly increases the risk of cholera transmission [32,55]. In Dadaab refugee camp in Kenya, Golicha et al. documented that the worst cholera outbreak since 1992 was linked to its proximity to bustling food markets where individuals from regions affected by cholera frequently gathered [50]. Some included studies in this review also reported that person-to-person contact, and overcrowding exacerbated the outbreaks [31,64,80]. A systematic review in India shed light on this mode of transmission and revealed that *V. cholerae* can be transmitted by coming into contact with cholera patients or asymptomatic carriers [3]. It has been explained that transmission often occurs through fomites, food, or water within households [3]. Without proper hygiene practices, such as handwashing, people who come into contact with these contaminated surfaces unknowingly spread the bacteria, further perpetuating the cholera outbreak via the fecal–oral route [3,119].

To control the spread of the disease during outbreaks, the Global Task Force on Cholera Control (GTFCC) advocates for the swift distribution of emergency WASH (water, sanitation, and hygiene) resources, such as water purification devices and emergency latrines, along with deployment of rapid response teams equipped with the case-area targeted intervention (CATI) strategy [120,121,122]. CATI targets the early identification of cholera clusters to enable a focused response within a high-risk radius around affected households, aiming to quickly reduce transmission and contain the outbreak [89,121]. 

In this review, we found that the overall detection rate of laboratory-confirmed cholera among suspected cases was 57.8% (95% CI: 49.2–66.4). Interestingly, Africa had a higher confirmation rate (62.9%) than Asia (45.6%), although the number of suspected cases was higher in Asia. This difference can be attributed to the greater number of outbreaks documented in Africa in this review. Cholera outbreaks are typically defined by the presence of at least one confirmed case of cholera along with evidence of local transmission [123]. Once an outbreak is declared, it is no longer necessary to confirm every suspected case; hence, a clinical case definition is considered sufficient for tracking epidemiological trends. However, for an outbreak to be officially declared, *V. cholerae* O1 or O139 must first be confirmed either by culture or PCR [123].

Among the more than 200 known serogroups of *V. cholerae*, only O1 and O139 have been consistently linked to cholera epidemics [4]. In this study, *V. cholerae* O1 emerged as the predominant serogroup. This dominance is largely due to the El Tor biotype, which can thrive in diverse environmental settings and cause asymptomatic infections, allowing cholera outbreaks to persist and spread over time [4,124]. The current cholera pandemic has been driven primarily by the El Tor biotype, which has effectively displaced the classical biotype, now considered extinct since its last recorded occurrence in the 1980s [125]. As expected, all included studies that reported the biotype confirmed the presence of El Tor.

The O1 strains are classified into three serotypes: Ogawa, Inaba, and Hikojima, which differ according to the methylation of the terminal perosamine in their oligosaccharide structures [124]. Ogawa, which is methylated, was responsible for 76.1% of cholera outbreaks, whereas Inaba, which is an unmethylated form, accounted for 15.2%. Ogawa and Inaba serotypes often co-circulate during epidemics and can interconvert [4,126]. This was observed in some studies that reported outbreaks (0.065%) with both serotypes existing [17,105]. This antigenic switching enables *V. cholerae* to evade immune detection and sustain transmission [4]. 

*V. cholerae* O139, which appeared in the early 1990s, has largely been confined to South and Southeast Asia [4,124]. Nevertheless, we observed its presence in outbreaks in Kenya and Uganda, indicating possible geographic expansion. Additionally, we observed one case of non-O1, non-O139 *Vibrio cholerae* (NOVC) associated with an outbreak [14]. Although NOVC strains do not produce cholera toxins and do not cause cholera, studies suggest that they account for between 1% and 3.4% of acute diarrheal cases worldwide [127,128].

Eliminating cholera has been a global priority, and the GTFCC introduced the “Ending Cholera: The Global Roadmap to 2030” strategy [129]. This framework envisions a world in which cholera no longer poses a public health threat, aiming to reduce cholera deaths by 90% and eliminate the disease in 20 countries [129]. We observed the pooled fatality prevalence during cholera outbreaks to be 1.3% and 1.3% (95% Cl: 1.1–1.6). This exceeds the minimum acceptable standard of less than 1% and reflects shortcomings in healthcare access and case management during outbreaks. Specifically, Africa had a significantly higher CFR (1.5%) than Asia (0.3%). CFR is a key indicator of healthcare quality during cholera outbreaks, with lower rates suggesting better access to treatment and timely interventions [130]. In Africa, limited resources, underfunded healthcare systems, and logistical challenges in providing timely treatment likely contribute to higher mortality [131,132].

Cholera treatment focuses on several key agents, including oral rehydration therapy (ORT), intravenous fluid therapy, antibiotics, and, in some cases, oral cholera vaccines (OCVs) [133]. The primary goal of this approach is to rehydrate patients and prevent dehydration, which is the leading cause of death in cholera cases [134,135]. The most widely recommended treatment is ORT, which involves administering a solution of salts and glucose to replace lost fluids and electrolytes [136,137]. In severe cases of extreme dehydration, intravenous fluids may be required to restore hydration rapidly [138].

Antibiotic therapy is another important treatment, particularly for patients with severe cholera. Although the World Health Organisation (WHO) recommends using antibiotics only for severe dehydration, the International Centre for Diarrhoeal Disease Research (ICDDR, B) recommends broader use, including for patients with moderate dehydration who continue to pass large amounts of diarrhea despite rehydration therapy [139]. Antibiotic therapy not only reduces illness duration but also limits transmission. Effective antibiotics can reduce the period during which patients shed *V. cholerae* from five or more days to just one or two days [139,140]. In a systematic review of antimicrobial drugs against *V. cholerae*, antimicrobial therapy was found to shorten the duration of diarrhea by about 1.5 days, reduce stool volume by 50%, and decrease the need for rehydration fluids by 40% [140]. The use of antibiotics not only accelerates recovery but also reduces the volume of rehydration fluids required and shortens the duration of hospitalization [133]. Studies have demonstrated the effectiveness of antibiotics like doxycycline and azithromycin, which can dramatically reduce diarrhea within 24 h, allowing patients to recover faster and leave treatment centers earlier [139,141].

One drawback to antibiotic therapy is that *the V. cholerae* O1 and O139 strains have developed resistance to most of the antibiotics that are used [142]. In this review, while most of the isolates showed susceptibility to antibiotics like ofloxacin, levofloxacin, norfloxacin, cefuroxime, doxycycline, and azithromycin, most of the isolates were resistant to traditional first-line antibiotics like ampicillin, cotrimoxazole, and amikacin. Most isolates also showed resistance to sulfamethoxazole, trimethoprim, furazolidone, and nalidixic acid.

To reduce the antimicrobial resistance of *V. cholerae*, the GTFCC provides specific guidance for selecting antibiotics during cholera outbreaks [123]. According to their recommendations, antibiotics should be selectively targeted to patients most likely to benefit clinically. The selection of antibiotics must be guided by current evidence on the sensitivity of circulating cholera strains, with regular monitoring for evolving resistance during outbreaks where feasible. Single-dose regimens are highly preferred over multi-dose treatments to facilitate easier implementation, particularly during large-scale outbreaks, and factors such as availability, cost, and ease of use are also important considerations when selecting an antibiotic [123]. The WHO advocates for the use of OCVs, particularly in endemic areas and during outbreaks [133,143]. When combined with ORT and antibiotics, OCVs provide temporary immunity and help reduce transmission risks [144].

We acknowledge some limitations in this review. The conclusions were primarily derived from peer-reviewed articles on cholera outbreaks, which may not fully reflect the actual incidence of outbreaks because of heterogeneity in reporting standards across regions. Moreover, we were unable to assess seasonal trends during the outbreaks, which could have enhanced our understanding of the epidemiology of the disease.

## 5. Conclusions

Cholera persists in impoverished areas, particularly in regions where crises weaken access to clean water and sanitation. Though treatments like doxycycline and azithromycin help manage such cases, the long-term solution lies in prevention. We advocate for the targeted deployment of cholera vaccines and the provision of safe drinking water through the chlorination of water sources and regular disinfection of tube wells. Moreover, public education on hygiene and upgrading of sewage systems is key to controlling outbreaks. Continuous surveillance will also ensure prompt action during future outbreaks.

## Data Availability

The original contributions presented in the study are included in the article/Supplementary Material, further inquiries can be directed to the corresponding author.

## Supplementary Materials

The following supporting information can be downloaded at: https://www.mdpi.com/article/10.3390/microorganisms12122504/s1, Table S1: PRISMA Checklist [145]; Table S2: Search Strategy; Table S3: Risk of Bias Assessment of Included Studies; Figure S1: Funnel Plot of Studies on Fatality Cases.

## Appendix A

**Table A1:** Study characteristics of included studies.

Study	Country	Region	Location	Study Design/Type	Outbreak Occurrence	Population at Risk	No. of Cases	CFR (No. of Deaths)
Abou et al., 2024 [12]	Lebanon	Africa	_	Descriptive	6 October 2022 to June 2023	_	8007	11% (23 deaths)
Abu Bashar et al., 2022 [13]	India	Asia	Nakhrauli Village, Ambala District	Descriptive	20 July 2018	1300	118	2 deaths
Amadu et al., 2021 [14]	Nigeria	Africa	Ilorin Metropolis	Retrospective descriptive	January to September 2017	_	5138	136 deaths
Alzain et al., 2021 [15]	Sudan	Africa	Northern State	Descriptive	February and September 2017	675,481	957	1.9%
Arnauot et al., 2024 [16]	Syria	Asia	_	Prospective observational cohort study	20 September to 20 October 2022	1712	1061	0.2%
Awuor et al., 2020 [17]	Kenya	Africa	Kisumu County	Descriptive (Cross-sectional)	January to December 2017	_		_
Bagcchi et al., 2022 [18]	Malawi	Africa	_	Descriptive (Outbreak report)	3 March 2022	_	78	5.1% (4 deaths)
Benamrouche et al., 2022 [19]	Algeria	Africa	Bouira, Blida, Algiers, Tipaza, Aïn Defla, Médéa and Oran	Descriptive	August 14 to 27 September 2018	_	291	_
Berhe et al., 2024 [20]	Ethiopia	Africa	Guraghe Zone	Descriptive	August 7 to 30 October 2023	234,161	224	2.6% (6 deaths)
Bitew et al., 2024 [21]	Ethiopia	Africa	Oromia National Regional State, Amhara National Regional State, Addis Ababa City	Descriptive (Cross-sectional)	26 September to 31 October 2023	_		_
Bompangue et al., 2020 [22]	Democratic Republic of the Congo	Africa	Kinshasa	Descriptive analysis (Cross-sectional)	January 2017 to November 2018	12 million	1712	_
Breurec et al., 2021 [23]	Central African Republic	Africa	_	Descriptive	July to Dec 2016	_	265	20 deaths
Bwire et al., 2023 [24]	Uganda	Africa	Bududa District	Cross-sectional	June to August 2019	259,800	67	1.5% (1 death)
Camacho et al., 2018 [25]	Yemen	Asia	_	Descriptive	28 September 2016 to 12 March 2018	29,932,971	1,103,683	0.22 (2385)
Cambaza et al., 2019 [26]	Mozambique	Africa	Cabo Delgado Province	Descriptive	2 May 2019	_	149	0
Chaguza et al., 2024 [27]	Malawi	Africa	_	Descriptive	2022 to 2023	_	59,156	3% (1759 deaths)
Challa et al., 2022 [28]	Uganda	Africa	Erer District	Descriptive (Case–control)	4 September to 1 November 2019	69,129	110	_
Chibwe et al., 2020 [29]	Malawi	Africa	Nsanje and Chikwawa Districts	Descriptive	March 2018	_	252	4 deaths
Chirambo et al., 2016 [30]	Zambia	Africa	Chibombo District	Descriptive (Cross-sectional)	9 February to 20 March 2016	5688	23	_
Dan-Nwafor et al., 2019 [31]	Nigeria	Africa	Gomali, Kwali Local Government Area	Descriptive (Case–control)	October–November 2014	1000	_	1.3 (6 deaths)
Davis et al., 2023 [32]	Ethiopia	Africa	Jijiga	Descriptive (Case–control)	1 January to 10 July	_	1000	_
Denue et al., 2018 [33]	Nigeria	Africa	Borno State	Descriptive	14 August 2017	1,489,638	5889	0.87% (43 deaths)
Dinede et al., 2020 [34]	Ethiopia	Africa	Addis Ababa	Descriptive (Case–control)	7 September 2017 to 1 October 2017	_	25	0
Dureab et al., 2019 [35]	Yemen	Asia	Aden	Descriptive (Case–control)	1 January to 31 March 2018	_	_	_
Dutta et al., 2021 [36]	India	Asia	Ghughri	Descriptive (Case–control)	18 August 2016	_	628	2% (14 deaths)
Eibach et al., 2016 [37]	Ghana	Africa	_	Descriptive	20 May 2014	_	20,120	0.8% (165 deaths)
Elfaki et al., 2023 [38]	Sudan	Africa	Blue Nile State	Descriptive	2018	1250	21	0
Elimian et al., 2020 [39]	Nigeria	Africa	_	Descriptive (Cross-sectional)	1 January–Nov ember 19	_	41,394	1.97% (2 deaths)
Emmanuel et al., 2019 [40]	Zimbabwe	Africa	Harare City	Descriptive (Cross-sectional)	5–25 September 2018	_	3564	0.9% (31 deaths)
Endris et al., 2019 [41]	Ethiopia	Africa	Addis Ababa	Descriptive	8 June to 31 October 2016	3,435,028	8083	0.18% (15 deaths)
Eurien et al., 2021 [42]	Uganda	Africa	Kampala City, Sembule Village	Descriptive (Case–control)	28 December 2018 to 11 February 2019	4700	50	6% (4 deaths)
Eyu et al., 2022 [43]	Uganda	Africa	Nebbi District	Descriptive (Case–control)	10 February 2017	409,000	222	1.35% (3 deaths)
Fagbamila et al., 2023 [44]	Nigeria	Africa	Bauchi State	Descriptive (Case–control)	28 February to 31 March 2019	4,653,066	9725	0.3% (28 deaths)
Faruque et al., 2021 [45]	Bangladesh	Asia	-	Descriptive (Cross-sectional)	September–December 2019	_	368	_
Feglo et al., 2018 [46]	Ghana	Africa	-	Descriptive	2014	_	28,975	243 deaths
George et al., 2018 [47]	Bangladesh	Asia	Mathbaria and Bakerganj	Descriptive (Cross-sectional)	April 2015 to June 2016	_	1081	_
Gopalkrishna et al., 2019 [48]	India	Asia	Aurangabad	Descriptive (Case–control)	10 November 2017	16,000	7447	_
Goswami et al., 2019 [49]	India	Asia	Wardha	Descriptive (Cross-sectional)	July	104	28	0
Golicha et al., 2018 [50]	Kenya	Africa	Dadaab Refugee Camp	Case–control	18 November 2015–6 June 2016	340,000	1797	0.79% (14 deaths)
Githuku et al., 2016 [51]	Kenya	Africa	Homa Bay County	Descriptive	January to April 2015	_	11,033	1.6% (178 deaths)
Grandesso et al., 2019 [52]	Malawi	Africa	Lake Chilwa	Descriptive (Case–control)	March to June 2016	_	236	0 deaths
Helou et al., 2023 [53]	Lebanon	Africa	_	Outbreak report	October to December 2022	_	5105	23 deaths
Iramiot et al., 2019 [54]	Uganda	Africa	Kasese District	Descriptive (Cross-sectional)	September 2017 to January 2018	757,269	222	1.4 (3 deaths)
Issahaku et al., 2020 [55]	Ghana	Africa	Central Region	Descriptive (Case–control)	12 October 2016 to 4 January 2017	160,849	704	0
Jain et al., 2021 [56]	India	Asia	Shahpur Huts, Panchkula District, Haryana State	Descriptive (Case–control)	1–28 September 2019	_	196	1% (2 deaths)
Jikal et al., 2019 [57]	Malaysia	Asia	Sabah	Descriptive (Case–control)	20 December 2014	_	78	_
Jones et al., 2020 [58]	South Sudan	Africa	_	Descriptive	2014–2017	11,654	28,645	622 deaths
Junejo et al., 2023 [59]	Pakistan	Asia	Karachi	Descriptive	2022	_	129	_
Kanu et al., 2018 [60]	Nigeria	Africa	Andoni, Rivers State	Descriptive (Case–control)	January 2015	211,009	1034	1.84% (19 deaths)
Kapata et al., 2018 [61]	Zambia	Africa	_	Descriptive	October 2017–February 2018	_	3938	2.1% (82 deaths)
Kaponda et al., 2019 [62]	Malawi	Africa	Karonga District	Descriptive (Cross-sectional)	November 2017 and March 2018	365,000	347	1.2% (4 deaths)
Kateule et al., 2024 [63]	Zambia	Africa	Lusaka District	Descriptive	18 October 2023–February 2024	2,248,140	13,122	2.8% (368 deaths)
Kisera et al., 2020 [64]	Kenya	Africa	Kakuma and Kalobeyei	Descriptive (Cross-sectional)	May 2017–2018	185,543		0.8% (1 death)
Kumar et al., 2022 [65]	India	Asia	Rahude Village, Nashik, Maharashtra	Descriptive	8–13 July 2018	850	195	1.03% (2 deaths)
Kigen et al., 2020 [66]	Kenya	Africa	Nairobi County	Descriptive (Case–control)	December 2014–June 2015	4,232,087	4218	1.9% (79 deaths)
Kwesiga et al., 2017 [67]	Uganda	Africa	Kasese District	Descriptive (Case–control)	May 2015	702,029	183	2 deaths
Madulla et al., 2023 [68]	Tanzania	Africa	Mwanza City	Descriptive (Cross-sectional)	August 2015–April 2016	706,453	852	0.80 (7 deaths)
Matapo et al., 2016 [69]	Zambia	Africa	Lusaka	Descriptive (Case–control)	February and May 2016	29,843	441	0.9 (4 deaths)
Mashe et al., 2020 [70]	Zimbabwe	Africa	_	Descriptive	4 September 2018 to 12 March 2019	6,148,243	10,730	0.6% (69 deaths)
Matimba et al., 2022 [71]	Tanzania	Africa	-	Descriptive (Cross-sectional)	August 2015 to December 2017	_	28,089	1.6% (452 deaths)
Mbala-Kingebeni et al., 2021 [72]	Democratic Republic of the Congo	Asia	Kinshasa	Descriptive (Case–control)	November 2017 to March 2018	_	_	_
McCrickard et al., 2017 [73]	Tanzania	Africa	Dar es Salaam	Descriptive	15 August 2015 to 31 October 2015	_	3371	1.1% (36 deaths)
Monje et al., 2020 [74]	Uganda	Africa	Kyangwali Refugee Settlement, Hoima District	Descriptive (Case–control)	1 February to 9 May 2019	668,900	2122	2.1% (44 deaths)
Mukhopadhyay et al., 2019 [75]	India	Asia	Kolkata Metropolis	Descriptive (Cross-sectional)	August 2015	_	3003	_
Mutale et al., 2020 [76]	Zambia	Africa	Lusaka	Descriptive (Case–control)	6 October 2017	4853	661	3% (20 deaths)
Mwaba et al., 2018 [77]	Zambia	Africa	Lusaka	Descriptive	4 February to 15 June 2016	2.3 million	251	_
Mwape et al., 2020 [78]	Zambia	Africa	Lusaka	Descriptive	February to June 2016	_	>1000	22 deaths
Mwenda et al., 2017 [79]	Kenya	Africa	Hotel in Nairobi	Descriptive	20 June 2017	456	456	_
Nanzaluka et al., 2020 [80]	Zambia	Africa	Lusaka	Descriptive (Case–control)	6 October 2017	_	1462	2.6% (38 deaths)
Neamin et al., 2020 [81]	Ethiopia	Africa	_	Descriptive	2015 to 2017	9,653,3716	36,154	0.7% (246 deaths)
Ngere et al., 2024 [82]	Kenya	Africa	Nairobi County	Descriptive (Cross-sectional)	2017	4,697,274	2737	1.5% (35 deaths)
Ngere et al., 2022 [83]	Kenya	Africa	_	Retrospective cohort study	31 August 2017 to 6 September 2017	526	139	0.7% (1 death)
Ngwa et al., 2020 [84]	Nigeria	Africa	Borno State	Descriptive (Cross-sectional)	August 2017	_	5340	1.14% (61 deaths)
Noora et al., 2017 [85]	Ghana	Africa	Brong Ahafo Region	Descriptive	July to December 2014	2.6 million	1035	0.024
Nsubuga et al., 2019 [86]	Sudan	Africa	Tonj East and Tonj North	Descriptive	1 May to 15 October 2017	412,969	1451	3.0% (44 deaths)
Oguttu et al., 2017 [87]	Uganda	Africa	Kaiso Village, Hoima District	Descriptive (Case–control)	1 October to 2 November 2015	9000	122	2 deaths
Ohene-Adjei et al., 2017 [88]	Ghana	Africa	_	Descriptive (Cross-sectional)	June 2014 to January 2015	4,530,905	20,199	0.6% (121 deaths)
Okeeffe et al., 2024 [89]	Nigeria	Africa	Borno, Adamawa, and Yobe States	Prospective observational cohort study	September to December 2021	1,538,000	111,062	3.2% (3604 deaths)
Okello et al., 2019 [90]	Uganda	Africa	Bulambuli District	Descriptive (Case–control)	1 March 2016	_		_
Pande et al., 2018 [91]	Uganda	Africa	Kasese District	Descriptive (Case–control)	20 June 2015	12324	61	_
Patel et al., 2022 [92]	India	Asia	Nadiad City	Descriptive	25 June 2021	2,540,037	158	0.63% (1 death)
Qaserah et al., 2021 [93]	Yemen	Asia	Al Hudaydah	Descriptive (Case–control)	December 1 2017 to January 10 2018	400,000	88,741	244 deaths
Roobthaisong et al., 2017 [94]	Myanmar	Asia	Mandalay	Descriptive	2015	_	1617	_
Roy et al., 2024 [95]	India	Asia	Dangapara Village, West Bengal	Descriptive	27 March 2021 to 8 April 2021	330	95	0
Sabir et al., 2023 [96]	Iraq	Asia	Sulaymaniyah Province	Descriptive	June–July 2022	_	4754	_
Shah et al., 2022 [97]	India	Asia	Gujarat	Descriptive	2015 to 2016	_	437	1.1% (1 death)
Sinyange et al., 2018 [98]	Zambia	Africa	Lusaka	Descriptive	October 2017–May 2018	_	5414	1.8 (98 deaths)
Sule et al., 2017 [99]	Nigeria	Africa	Kaduna State	Descriptive	2014	7.685 million	1468	3.68 (54 deaths)
Ujjiga et al., 2015 [101]	South Sudan	Africa	_	Descriptive (Case–control)	23 April 2014	_	2260	2.0% (43 deaths)
Wang et al., 2016 [102]	Tanzania	Africa	_	Descriptive	August 2015 to June 2016	_	21,750	341 deaths
Winstead et al., 2020 [103]	Zimbabwe	Africa	Harare Province	Descriptive	September 2018–March 2019	_	10,730	0.64% (69)
Zahid et al., 2022 [104]	Pakistan	Asia	Karachi	Observational	March to May 2022	_	_	_
Zgheir et al., 2019 [105]	Iraq	Asia	_	Descriptive	August 2017	_	1367	0.6% (30 deaths)
Zhao et al., 2019 [106]	Yemen	Asia	_	Descriptive	May 2017 to 2018	_	111,3578	2310 deaths

**Table A2:** Causes of cholera outbreaks.

Study	Risk Factors Assessed	Transmission Route/Suspected Exposure	Serogroup	Serotype/Biotype	Number Examined	Number Infected
Abou et al., 2024 [12]	-	-	*V. cholerae* O1	Ogawa/ El Tor	-	671
Abu Bashar et al., 2022 [13]	Water source (filtration method or purifier, storage method), history of travel, mass gathering attendance	Waterborne	*V. cholerae* O1	Ogawa/ El Tor	3	1
Amadu et al., 2021 [14]	-	-	*V. cholerae* O1	Inaba and non-O1/O139	102	42
Al Zain et al., 2021 [15]	-	Travel	-	-	-	-
Arnauot et al., 2024 [16]	Water source (well water, swimming pool), food	Waterborne, foodborne	-	-	-	--
Awuor et al., 2020 [17]	-	-	*V. cholerae* O1	Ogawa and Inaba/El Tor	119	101
Bagcchi et al., 2022 [18]	-	-	*V. cholerae* O1	Inaba	-	-
Benamrouche et al., 2022 [19]	-	-	*V. cholerae* O1	Ogawa/ El Tor	291	91
Berhe et al., 2024 [20]	-	Poor hygiene and sanitation	-		-	-
Bitew et al., 2024 [21]	Water source, outbreak season, toilet use frequency	Waterborne	*V. cholerae* O1	Ogawa	-	-
Bompangue et al., 2020 [22]	-	Waterborne, poor hygiene and sanitation	-	-	-	-
Breurec et al., 2021 [23]	-	Waterborne, poor hygiene and sanitation, overcrowding, lack of latrines	*V. cholerae* O1	Inaba/ El Tor	-	-
Chaguza et al., 2024 [27]	-	Cyclone Gombe	*V. cholerae* O1	Ogawa/ El Tor	44	42
Challa et al., 2022 [28]	Water source, eating habits, hygiene and sanitation	Poor hygiene and sanitation	-	-	-	-
Chibwe et al., 2020 [29]	-	Flood	*V. cholerae* O1	Ogawa	80	57
Chirambo et al., 2016 [30]	-	Waterborne, poor sanitation, inadequate sanitary facilities	-	-	18	8
Dan-Nwafor et al., 2019 [31]	Water source, hygiene and sanitation, >5 persons per household, No formal education, Lack of rack for drying plates	Waterborne, poor personal hygiene, overcrowding	-	-	4	4
Davis et al., 2023 [32]	Water source, eating habits, hygiene and sanitation	Waterborne	-	-	-	-
Denue et al., 2018 [33]	-	Heavy rain, flooding	*V. cholerae* O1	Ogawa	-	-
Dinede et al., 2020 [34]	Water source, eating habits, hygie and sanitation	Waterborne, foodborne	-		7	-
Dureab et al., 2019 [35]	Travel history, water source, eating habits, hygiene and sanitation	Foodborne, contact	-	-	-	-
Dutta et al., 2021 [36]	Water source, defecation practices, and toilet facilities	Heavy rain, open defecation, waterborne	*V. cholerae* O1	Ogawa	34	11
Eibach et al., 2016 [37]	-	Rain	*V. cholerae* O1	Ogawa/ El Tor	496	264
Emmanuel et al., 2019 [40]	-	-	-	-	83	-
Endris et al., 2019 [41]	Travel history, water source, contact	Foodborne, waterborne, open defecation	-		-	-
Eurien et al., 2021 [42]	-	-	*V. cholerae* O1	Ogawa	45	22
Eyu et al., 2022 [43]	-	Waterborne	*V. cholerae* O139	-	5	5
Fagbamila et al., 2023 [44]	Attending social gatherings, source of drinking water, eating habits, hygiene and sanitation	Waterborne, attending social gathering	-	-	-	-
Faruque et al., 2021 [45]	Water source, toilet use pattern	-	-		-	-
Feglo et al., 2018 [46]	-	-	*V. cholerae* O1	Ogawa/ El tor	62	40
George et al., 2018 [47]	Contact, wating habits, hygiene and sanitation	Waterborne, foodborne, poor sanitation	*V. cholerae* O1	Ogawa	51	30
Gopalkrishna et al., 2019 [48]	Water source	Waterborne	*V. cholerae* O1	Ogawa/ El tor	46	6
Goswami et al., 2019 [49]	Source of drinking water	Waterborne, travel history	*V. cholerae* O1	Ogawa/ El tor	2	2
Golicha et al., 2018 [50]	Water sources, hygiene and sanitation, eating habits	Waterborne, attending social gathering	*V. cholerae* O1	Ogawa	-	-
Githuku et al., 2016 [51]	-	-	*V. cholerae* O1	Ogawa/ El Tor	-	-
Grandesso et al., 2019 [52]	Contact, water source, food consumption, type of toilet used, hygiene and sanitation,	-	*V. cholerae* O1		151	116
Helou et al., 2023 [53]	-	-	V. cholerae O1	-	-	-
Iramiot et al., 2019 [54]	Water sources and safety, hygiene and sanitation	Open defecation	*V. cholerae* O1	Inaba	71	69
Issahaku et al., 2020 [55]	Visit to the CTC, wash hands before eating, eating street-vended food, drinking sachet water	Visiting cholera treatment centers	*V. cholerae* O1	Ogawa	-	-
Jain et al., 2021 [56]	Water from tanks, sewage effluent	Waterborne	-		18	4
Jikal et al., 2019 [57]	Water sources, hygiene and sanitation, eating habits	Foodborne	*V. cholerae* O1	Ogawa/El tor	-	-
Jones et al., 2020 [58]	-	Waterborne	*V. cholerae* O1		964	372
Junejo et al., 2023 [59]	Water sources	Poor sanitation	*V. cholerae* O1	Ogawa	-	-
Kanu et al., 2018 [60]	Water sources, hygiene and sanitation, contact, eating habits	-	-	-	-	-
Kapata et al., 2018 [61]	-	Waterborne	-		-	-
Kaponda et al., 2019 [62]	Source drinking water	Waterborne	-		-	-
Kateule et al., 2024 [63]	-	Heavy rain, waterborne	*V. cholerae* O1	Ogawa	2091	253
Kisera et al., 2020 [64]	-	overcrowding, poor sanitation	*V. cholerae* O1	Inaba	30	22
Kumar et al., 2022 [65]	-	Waterborne, open defecation	*V. cholerae* O1	Ogawa	-	-
Kigen et al., 2020 [66]	Water sources, contact, hygiene and sanitation, education, eating habits	-	-	-	-	-
Kwesiga et al., 2017 [67]	Water sources, hygiene and sanitation, eating habits	Waterborne	*V. cholerae* O1	Inaba/ El Tor	78	61
Madulla et al., 2023 [68]	Water source	Waterborne	-		452	352
Matapo et al., 2016 [69]	Contact, access to toilet facility, knowledge on cholera, water source, hygiene and sanitation, eating habits	Waterborne	*V. cholerae* O1	Ogawa	-	-
Mashe et al., 2020 [70]	-	-	*V. cholerae* O1	Ogawa	371	241
Matimba et al., 2022 [71]	-	-	*V. cholerae* O1	Ogawa	99	97
Mbala-Kingebeni et al., 2021 [72]	Water sources, hygiene and sanitation, eating habits	Waterborne	-	-	177	83
McCrickard et al., 2017 [73]	-	-	*V. cholerae* O1	-	56	39
Monje et al., 2020 [74]	Water sources	Waterborne	-	-	130	124
Mukhopadhyay et al., 2019 [75]	Water sources	-	*V. cholerae* O1	-	204	63
Mwaba et al., 2018 [77]	-	-	*V. cholerae* O1	Ogawa	170	62
Mwape et al., 2020 [78]	-	-	*V. cholerae* O1	Ogawa	52	52
Mwenda et al., 2017 [79]	-	Foodborne	*V. cholerae* O139	Ogawa/ El Tor	24	18
Nanzaluka et al., 2020 [80]	Water sources, eating habits, hygiene and sanitation, household member with cholera, household shares latrine, cholera vaccination	Waterborne, contact	-	-	-	-
Neamin et al., 2020 [81]	-	-	-	-	4477	4054
Ngere et al., 2024 [82]	Mass gathering attendance	Attending social gathering	*V. cholerae* O1	Ogawa	224	78
Ngere et al., 2022 [83]	-	Foodborne	*V. cholerae* O1	Ogawa	69	7
Noora et al., 2017 [85]	-	Foodborne	*V. cholerae* O1	-	-	-
Nsubuga et al., 2019 [86]	-	-	*V. cholerae* O1	Inaba	25	21
Oguttu et al., 2017 [87]	Water source	Waterborne	-	-	10	6
Ohene-Adjei et al., 2017 [88]	-	Waterborne, foodborne, poor hygiene and sanitation, open defecation	-		-	-
Okello et al., 2019 [90]	Water source	Waterborne	-	-	-	-
Pande et al., 2018 [91]	Water source	Waterborne	-	-	61	8
Qaserah et al., 2021 [93]	Water sources, hygiene and sanitation, household member with cholera, household shares latrine, eating habits	Waterborne	-	-	-	-
Roobthaisong et al., 2017 [94]	-	-	*V. cholerae* O1	66 were Ogawa/El Tor, 1 was Inaba	67	67
Roy et al., 2024 [95]	Water source, open defecation practices, public gathering attendance, eating habits, hygiene and sanitation	Waterborne	-		-	-
Sabir et al., 2023 [96]	-	-	*V. cholerae* O1	Ogawa	414	414
Shah et al., 2022 [97]	-	Waterborne	*V. cholerae* O1	Ogawa/El Tor	5	3
Sinyange et al., 2018 [98]	Water source	-	*V. cholerae* O1	Ogawa/El Tor	2054	925
Sule et al., 2017 [99]	-	-	-	-	22	2
Ujjiga et al., 2015 [101]	-	Wars	-	-	6175	25
Wang et al., 2016 [102]	Eating habits, travel outside home village, treated drinking water at home, had 2 oral cholera vaccine doses	Foodborne, travel	-		-	-
Winstead et al., 2020 [103]	-	-	*V. cholerae* O1		-	-
Zahid et al., 2022 [104]	-	-	*V. cholerae* O1	Ogawa	378	78
Zgheir et al., 2019 [105]	Water source	Waterborne	*V. cholerae* O1	98.4% Inaba/ElTor, 1.58% Ogawa/El Tor	1367	505
Zhao et al., 2019 [106]	-	War, waterborne	-		-	-
