# Supplementary material for: Cholera Outbreaks in Low- and Middle-Income Countries in the Last Decade: A Systematic Review and Meta-Analysis
Source: Microorganisms. 2024 Dec 4;12(12):2504. doi: 10.3390/microorganisms12122504 (PMC11728267; doi:10.3390/microorganisms12122504)
Supplement: Supplementary file 1 [file microorganisms-12-02504-s001.zip › Table_S2_(Search_Strategy).pdf]

# Cholera Outbreaks in Low-and Middle-Income Countries in the Last Decade: A Systematic Review

## Supplementary Table S2. Search Strategy

### Last Search Date: September 21, 2024

| # | Database | Query String | Records Found |
|---|---|---|---|
| 1 | **PubMed** | ("Cholera infection" OR "cholera outbreak") AND ("Afghanistan" OR "Korea, Dem. People's Rep" OR "South Sudan" OR "Burkina Faso" OR "Liberia" OR "Sudan" OR "Burundi" OR "Madagascar" OR "Syrian Arab Republic" OR "Central African Republic" OR "Malawi" OR "Togo" OR "Chad" OR "Mali" OR "Uganda" OR "Congo, Dem. Rep" OR "Mozambique" OR "Yemen, Rep" OR "Eritrea" OR "Niger" OR "Ethiopia" OR "Rwanda" OR "Gambia, The" OR "Sierra Leone" OR "Guinea-Bissau" OR "Somalia" OR "Angola" OR "Honduras" OR "Papua New Guinea" OR "Bangladesh" OR "India" OR "Philippines" OR "Benin" OR "Jordan" OR "Samoa" OR "Bhutan" OR "Kenya" OR "São Tomé and Principe" OR "Bolivia" OR "Kiribati" OR "Senegal" OR "Cabo Verde" OR "Kyrgyz Republic" OR "Solomon Islands" OR "Cambodia" OR "Lao PDR" OR "Sri Lanka" OR "Cameroon" OR "Lebanon" OR "Tajikistan" OR "Comoros" OR "Lesotho" OR "Tanzania" OR "Congo, Rep" OR "Mauritania" OR "Timor-Leste" OR "Côte d'Ivoire" OR "Micronesia, Fed. Sts" OR "Tunisia" OR "Djibouti" OR "Morocco" OR "Uzbekistan" OR "Egypt Arab Rep" OR "Myanmar" OR "Vanuatu" OR "Eswatini" OR "Nepal" OR "Vietnam" OR "Ghana" OR "Nicaragua" OR "West Bank and Gaza" OR "Guinea" OR "Nigeria" OR "Zambia" OR "Zimbabwe" OR "Albania" OR "Equatorial Guinea" OR "Moldova" OR "Algeria" OR "Fiji" OR "Mongolia" OR "Argentina" OR "Gabon" OR "Montenegro" OR "Armenia" OR "Georgia" OR "Namibia" OR "Azerbaijan" OR "Grenada" OR "North Macedonia" OR "Belarus" OR "Guatemala" OR "Paraguay" OR "Belize" OR "Indonesia" OR "Peru" OR "Bosnia and Herzegovina" OR "Iran, Islamic Rep" OR "Serbia" OR "Botswana" OR "Iraq" OR "South Africa" OR "Brazil" OR "Jamaica" OR "St. Lucia" OR "China" OR "Kazakhstan" OR "St. Vincent and the Grenadines" OR "Colombia" OR "Kosovo" OR "Suriname" OR "Costa Rica" OR "Libya" OR "Thailand" OR "Cuba" OR "Malaysia" OR "Tonga" OR "Dominica" OR "Maldives" OR "Türkiye" OR "Dominican Republic" OR "Marshall Islands" OR "Turkmenistan" OR "Ecuador" OR "Mauritius" OR "Tuvalu" OR "El Salvador" OR "Mexico" OR "Ukraine") | 193 |

| | | | |
|---|---|---|---|
| 2 | **Scopus** | TITLE-ABS-KEY ( ( "Cholera infection" OR "cholera outbreak" ) AND ( "Afghanistan" OR "Korea, Dem. People's Rep" OR "South Sudan" OR "Burkina Faso" OR "Liberia" OR "Sudan" OR "Burundi" OR "Madagascar" OR "Syrian Arab Republic" OR "Central African Republic" OR "Malawi" OR "Togo" OR "Chad" OR "Mali" OR "Uganda" OR "Congo, Dem. Rep" OR "Mozambique" OR "Yemen, Rep" OR "Eritrea" OR "Niger" OR "Ethiopia" OR "Rwanda" OR "Gambia, The" OR "Sierra Leone" OR "Guinea-Bissau" OR "Somalia" OR "Angola" OR "Honduras" OR "Papua New Guinea" OR "Bangladesh" OR "India" OR "Philippines" OR "Benin" OR "Jordan" OR "Samoa" OR "Bhutan" OR "Kenya" OR "São Tomé and Principe" OR "Bolivia" OR "Kiribati" OR "Senegal" OR "Cabo Verde" OR "Kyrgyz Republic" OR "Solomon Islands" OR "Cambodia" OR "Lao PDR" OR "Sri Lanka" OR "Cameroon" OR "Lebanon" OR "Tajikistan" OR "Comoros" OR "Lesotho" OR "Tanzania" OR "Congo, Rep" OR "Mauritania" OR "Timor-Leste" OR "Côte d'Ivoire" OR "Micronesia, Fed. Sts" OR "Tunisia" OR "Djibouti" OR "Morocco" OR "Uzbekistan" OR "Egypt Arab Rep" OR "Myanmar" OR "Vanuatu" OR "Eswatini" OR "Nepal" OR "Vietnam" OR "Ghana" OR "Nicaragua" OR "West Bank and Gaza" OR "Guinea" OR "Nigeria" OR "Zambia" OR "Zimbabwe" OR "Albania" OR "Equatorial Guinea" OR "Moldova" OR "Algeria" OR "Fiji" OR "Mongolia" OR "Argentina" OR "Gabon" OR "Montenegro" OR "Armenia" OR "Georgia" OR "Namibia" OR "Azerbaijan" OR "Grenada" OR "North Macedonia" OR "Belarus" OR "Guatemala" OR "Paraguay" OR "Belize" OR "Indonesia" OR "Peru" OR "Bosnia and Herzegovina" OR "Iran, Islamic Rep" OR "Serbia" OR "Botswana" OR "Iraq" OR "South Africa" OR "Brazil" OR "Jamaica" OR "St. Lucia" OR "China" OR "Kazakhstan" OR "St. Vincent and the Grenadines" OR "Colombia" OR "Kosovo" OR "Suriname" OR "Costa Rica" OR "Libya" OR "Thailand" OR "Cuba" OR "Malaysia" OR "Tonga" OR "Dominica" OR "Maldives" OR "Türkiye" OR "Dominican Republic" OR "Marshall Islands" OR "Turkmenistan" OR "Ecuador" OR "Mauritius" OR "Tuvalu" OR "El Salvador" OR "Mexico" OR "Ukraine" ) ) AND PUBYEAR > 2013 AND PUBYEAR < 2025 AND ( LIMIT-TO ( DOCTYPE , "ar" ) ) | 768 |
| 5 | **Web of Science** | ("Cholera infection" OR "cholera outbreak") AND ("Afghanistan" OR "Korea, Dem. People's Rep" OR "South Sudan" OR "Burkina Faso" OR "Liberia" OR "Sudan" OR "Burundi" OR "Madagascar" OR "Syrian Arab Republic" OR "Central African Republic" OR "Malawi" OR "Togo" OR "Chad" OR "Mali" OR "Uganda" OR "Congo, Dem. Rep" OR "Mozambique" OR "Yemen, Rep" OR "Eritrea" OR "Niger" OR "Ethiopia" OR "Rwanda" OR "Gambia, The" OR "Sierra Leone" OR "Guinea-Bissau" OR "Somalia" OR "Angola" OR "Honduras" OR "Papua New Guinea" OR "Bangladesh" OR "India" OR "Philippines" OR "Benin" OR "Jordan" OR "Samoa" OR "Bhutan" OR "Kenya" OR "São Tomé and Principe" OR "Bolivia" OR "Kiribati" OR "Senegal" OR "Cabo Verde" OR "Kyrgyz Republic" OR "Solomon Islands" OR "Cambodia" OR "Lao PDR" OR "Sri Lanka" OR "Cameroon" OR "Lebanon" OR "Tajikistan" OR "Comoros" OR "Lesotho" OR "Tanzania" OR "Congo, Rep" | 301 |

| | | | |
|---|---|---|---|
| | | OR "Mauritania" OR "Timor-Leste" OR "Côte d'Ivoire" OR "Micronesia, Fed. Sts" OR "Tunisia" OR "Djibouti" OR "Morocco" OR "Uzbekistan" OR "Egypt Arab Rep" OR "Myanmar" OR "Vanuatu" OR "Eswatini" OR "Nepal" OR "Vietnam" OR "Ghana" OR "Nicaragua" OR "West Bank and Gaza" OR "Guinea" OR "Nigeria" OR "Zambia" OR "Zimbabwe" OR "Albania" OR "Equatorial Guinea" OR "Moldova" OR "Algeria" OR "Fiji" OR "Mongolia" OR "Argentina" OR "Gabon" OR "Montenegro" OR "Armenia" OR "Georgia" OR "Namibia" OR "Azerbaijan" OR "Grenada" OR "North Macedonia" OR "Belarus" OR "Guatemala" OR "Paraguay" OR "Belize" OR "Indonesia" OR "Peru" OR "Bosnia and Herzegovina" OR "Iran, Islamic Rep" OR "Serbia" OR "Botswana" OR "Iraq" OR "South Africa" OR "Brazil" OR "Jamaica" OR "St. Lucia" OR "China" OR "Kazakhstan" OR "St. Vincent and the Grenadines" OR "Colombia" OR "Kosovo" OR "Suriname" OR "Costa Rica" OR "Libya" OR "Thailand" OR "Cuba" OR "Malaysia" OR "Tonga" OR "Dominica" OR "Maldives" OR "Türkiye" OR "Dominican Republic" OR "Marshall Islands" OR "Turkmenistan" OR "Ecuador" OR "Mauritius" OR "Tuvalu" OR "El Salvador" OR "Mexico" OR "Ukraine") (Topic) and 2014 or 2015 or 2017 or 2018 or 2016 or 2024 or 2023 or 2022 or 2021 or 2020 or 2019 (Publication Years) and Review Article (Exclude – Document Types) | |
| 6 | **Google Scholar** | cholera outbreak OR Vibrio cholerae OR cholera AND outbreak OR epidemic | First 400 results |
| | | **Total Records Found** | **1662** |
