# Supplementary material for: Cholera Outbreaks in Low- and Middle-Income Countries in the Last Decade: A Systematic Review and Meta-Analysis
Source: Microorganisms. 2024 Dec 4;12(12):2504. doi: 10.3390/microorganisms12122504 (PMC11728267; doi:10.3390/microorganisms12122504)
Supplement: Supplementary file 1 [file microorganisms-12-02504-s001.zip › Table_S3 (Risk_of_Bias).pdf]

Cholera Outbreaks in Low-and Middle-Income Countries in the Last Decade: A Systematic Review and Meta-Analysis

Supplementary Table S3. Risk of Bias Assessment of Included Studies

The risk of bias in the included studies was evaluated using a modified version of the Downes et al. critical appraisal tool (AXIS) [1], adjusted in line with the approach used by Muzembo et al.[2]. Key appraisal criteria included the clarity of study objectives, the robustness of study design descriptions, and the presence of a precise cholera definition. The evaluation also reviewed sampling methods, reporting of outcomes like risk factors and case fatality rates, as well as the rigor of data collection and statistical analysis. Additionally, the use of diagnostic methods (e.g., culture or PCR for cholera confirmation) and discussions of study limitations and potential confounders were assessed. Each criterion was scored as 1 for "yes" and 0 for "no" or "unclear." Studies were then categorized based on their total scores: 0–4 for high risk of bias, 5–7 for moderate risk, and 8–9 for low risk.

Study	Aim Clearly Stated	Setting Provided	Study Design or Sampling Method Explained	Case Definition of Diarrheal or Cholera Clearly Mentioned	Statistical or Analysis Methods Reported	Risk Factors for Outbreak (or Causes of Outbreaks) Investigated	Case Fatality Ratio/ Number of deaths Reported	Performance of Confirmatory Test (Culture or PCR)	Limitations or Potential Confounders Discussed	Score	Risk of Bias
Abou et al.	Yes	No	Yes	No	Yes	No	Yes	No	No	5	Moderate
Abu Bashar et al.	Yes	Yes	Yes	Yes	Yes	Yes	Yes	Yes	Yes	9	Low
Abubakar et al.	Yes	Yes	Yes	No	Yes	No	Yes	No	No	5	Moderate
Amadu et al.	Yes	Yes	Yes	No	Yes	No	No	Yes	No	5	Moderate
Al Zain et al.	Yes	Yes	Yes	Yes	Yes	No	Yes	No	Yes	7	Moderate
Arnauot et al.	Yes	Yes	Yes	Yes	Yes	Yes	Yes	No	Yes	8	Low
Awuor et al.	Yes	Yes	Yes	No	Yes	No	Yes	Yes	No	6	Moderate
Bagcchi et al.	No	No	No	No	No	No	Yes	No	No	2	High
Benamrouche et al.	Yes	No	Yes	No	No	No	Yes	Yes	No	5	Moderate
Berhe et al.	Yes	Yes	Yes	Yes	Yes	Yes	Yes	Yes	Yes	9	Low
Bitew et al.	Yes	Yes	Yes	No	Yes	Yes	Yes	Yes	No	7	Moderate
Bompangue	Yes	Yes	Yes	Yes	Yes	Yes	Yes	No	Yes	8	Low
Breurec et al.	Yes	Yes	Yes	No	Yes	No	Yes	Yes	No	6	Moderate
Bwire et al.	Yes	Yes	Yes	Yes	Yes	Yes	Yes	Yes	Yes	9	Low
Camacho et al.	Yes	No	Yes	Yes	Yes	No	Yes	Yes	No	9	Low
Cambaza et al.	Yes	Yes	Yes	No	No	No	Yes	No	No	7	Moderate
Chaguza et al.	Yes	No	Yes	Yes	No	No	Yes	Yes	No	4	High
Challa et al.	Yes	Yes	Yes	Yes	Yes	Yes	Yes	No	Yes	6	Moderate
Chibwe et al.	Yes	Yes	Yes	Yes	Yes	No	Yes	Yes	No	8	Low
Chirambo et al.	Yes	Yes	Yes	Yes	No	Yes	Yes	Yes	No	7	Moderate

Study	Aim Clearly Stated	Setting Provided	Study Design or Sampling Method Explained	Case Definition of Diarrheal or Cholera Clearly Mentioned	Statistical or Analysis Methods Reported	Risk Factors for Outbreak (or Causes of Outbreaks) Investigated	Case Fatality Ratio/ Number of deaths Reported	Performance of Confirmatory Test (Culture or PCR)	Limitations or Potential Confounders Discussed	Score	Risk of Bias
Dan-Nwafor et al.	Yes	Yes	Yes	Yes	Yes	Yes	Yes	Yes	Yes	7	Moderate
Davis et al.	Yes	No	Yes	Yes	Yes	Yes	No	No	Yes	9	Low
Denue et al.	Yes	Yes	Yes	Yes	Yes	No	Yes	No	Yes	7	Moderate
Dinede et al.	Yes	Yes	Yes	Yes	Yes	Yes	Yes	Yes	Yes	7	Moderate
Duread et al.	Yes	Yes	Yes	No	Yes	Yes	No	No	Yes	9	Low
Dutta et al.	Yes	Yes	Yes	Yes	Yes	Yes	Yes	Yes	Yes	6	Moderate
Eibach et al.	Yes	Yes	Yes	Yes	Yes	No	Yes	Yes	Yes	9	Low
Elimian et al.	Yes	Yes	Yes	Yes	Yes	No	Yes	No	Yes	8	Low
Emmanuel et al.	Yes	Yes	Yes	No	Yes	No	Yes	No	Yes	9	Low
Endris et al.	Yes	Yes	Yes	Yes	Yes	Yes	Yes	Yes	No	6	Moderate
Eurien et al.	Yes	Yes	Yes	Yes	Yes	Yes	Yes	Yes	Yes	8	Low
Eyu et al.	Yes	Yes	Yes	Yes	Yes	Yes	Yes	Yes	Yes	9	Low
Fagbamila et al.	Yes	Yes	Yes	Yes	yes	Yes	Yes	Yes	Yes	9	Low
Faruque et al.	Yes	Yes	Yes	No	Yes	Yes	No	Yes	Yes	9	Low
Feglo et al.	Yes	No	Yes	No	No	No	No	Yes	Yes	7	Moderate
George et al.	No	No	Yes	Yes	No	No	Yes	No	No	4	High
Gopalkrishna et al.	No	No	No	Yes	No	No	No	Yes	No	3	High

Study	Aim Clearly Stated	Setting Provided	Study Design or Sampling Method Explained	Case Definition of Diarrheal or Cholera Clearly Mentioned	Statistical or Analysis Methods Reported	Risk Factors for Outbreak (or Causes of Outbreaks) Investigated	Case Fatality Ratio/ Number of deaths Reported	Performance of Confirmatory Test (Culture or PCR)	Limitations or Potential Confounders Discussed	Score	Risk of Bias
Goswami et al.	Yes	Yes	Yes	Yes	Yes	No	No	Yes	No	6	Moderate
Golicha et al.	Yes	No	Yes	Yes	Yes	Yes	Yes	Yes	Yes	9	Low
Githuku et al.	No	No	No	No	No	No	Yes	Yes	No	3	High
Grandesso et al.	Yes	Yes	Yes	Yes	Yes	No	Yes	Yes	Yes	8	Low
Helou et al.	No	No	No	No	No	No	Yes	No	No	2	High
Iramiot et al.	Yes	Yes	Yes	Yes	Yes	M	Yes	Yes	Yes	8	Low
Issahaku et al.	Yes	Yes	Yes	Yes	Yes	Yes	Yes	Yes	Yes	9	Low
Jain et al.	Yes	Yes	Yes	Yes	No	No	Yes	Yes	Yes	7	Moderate
Jikal et al.	Yes	No	Yes	Yes	No	Yes	Yes	Yes	No	7	Moderate
Jones et al.	Yes	No	Yes	Yes	Yes	No	Yes	Yes	Yes	8	Low
Junejo et al.	Yes	No	Yes	No	Yes	No	No	Yes	Yes	6	Moderate
Kanu et al.	Yes	Yes	Yes	Yes	Yes	Yes	Yes	Yes	No	8	Low
Kapata et al.	Yes	No	No	No	No	No	Yes	No	No	3	High
Kaponda et al.	Yes	Yes	Yes	Yes	Yes	Yes	Yes	No	Yes	8	Low
Kateule et al.	Yes	Yes	Yes	No	Yes	Yes	Yes	Yes	No	7	Moderate
Kisera et al.	Yes	Yes	Yes	No	Yes	No	No	No	Yes	5	Moderate
Kumar et al.	Yes	Yes	Yes	Yes	Yes	Yes	Yes	No	Yes	8	Low
Kigen et al.	Yes	Yes	Yes	Yes	No	Yes	Yes	Yes	Yes	8	Low

**Study**	**Aim Clearly Stated**	**Setting Provided**	**Study Design or Sampling Method Explained**	**Case Definition of Diarrheal or Cholera Clearly Mentioned**	**Statistical or Analysis Methods Reported**	**Risk Factors for Outbreak (or Causes of Outbreaks) Investigated**	**Case Fatality Ratio/ Number of deaths Reported**	**Performance of Confirmatory Test (Culture or PCR)**	**Limitations or Potential Confounders Discussed**	**Score**	**Risk of Bias**
Kwesiga et al.	Yes	Yes	Yes	No	Yes	Yes	No	Yes	Yes	7	Moderate
Madulla et al.	Yes	Yes	Yes	No	Yes	Yes	Yes	Yes	Yes	8	Low
Matapo et al.	Yes	Yes	Yes	Yes	Yes	Yes	Yes	Yes	No	8	Low
Mashe et al.	Yes	Yes	Yes	Yes	No	No	Yes	Yes	Yes	7	Moderate
Matimba et al.	Yes	Yes	Yes	Yes	Yes	No	Yes	Yes	No	7	Moderate
Mbala-Kingebeni et al.	Yes	Yes	Yes	Yes	Yes	Yes	Yes	No	Yes	8	Low
McCrickard et al.	Yes	No	Yes	Yes	Yes	No	Yes	Yes	Yes	8	Low
Monje et al.	Yes	Yes	Yes	Yes	Yes	Yes	Yes	Yes	Yes	9	Low
Mukhopadhyay et al.	Yes	No	Yes	No	Yes	No	Yes	Yes	No	6	Moderate
Mutale et al.	Yes	No	Yes	Yes	Yes	Yes	Yes	No	Yes	8	Low
Mwaba et al.	Yes	Yes	Yes	Yes	Yes	No	No	Yes	Yes	7	Moderate
Mwape et al.	Yes	No	Yes	No	Yes	No	Yes	Yes	No	6	Moderate
Mwenda et al.	Yes	Yes	Yes	No	Yes	Yes	No	Yes	Yes	7	Moderate
Nanzaluka et al.	Yes	No	Yes	Yes	Yes	Yes	Yes	No	Yes	8	Low
Neamin et al.	Yes	Yes	Yes	Yes	Yes	No	Yes	No	Yes	7	Moderate
Ngere et al. 2024	Yes	Yes	Yes	Yes	Yes	Yes	Yes	Yes	Yes	9	Low
Ngere et al. 2022	Yes	Yes	Yes	Yes	Yes	Yes	Yes	Yes	Yes	9	Low
Ngwa et al.	Yes	Yes	Yes	No	No	No	Yes	No	No	4	High
Noora et al.	Yes	Yes	Yes	Yes	Yes	No	Yes	Yes	No	7	Moderate
Nsubuga et al.	Yes	Yes	Yes	Yes	Yes	No	Yes	Yes	Yes	8	Low

Study	Aim Clearly Stated	Setting Provided	Study Design or Sampling Method Explained	Case Definition of Diarrheal or Cholera Clearly Mentioned	Statistical or Analysis Methods Reported	Risk Factors for Outbreak (or Causes of Outbreaks) Investigated	Case Fatality Ratio/ Number of deaths Reported	Performance of Confirmatory Test (Culture or PCR)	Limitations or Potential Confounders Discussed	Score	Risk of Bias
Oguttu et al.	Yes	Yes	Yes	Yes	Yes	Yes	Yes	Yes	No	8	Low
Ohene-Adjei et al.	Yes	Yes	Yes	No	Yes	Yes	Yes	No	Yes	7	Moderate
Okeeffe et al.	Yes	Yes	Yes	No	Yes	Yes	Yes	No	Yes	7	Moderate
Okello et al.	Yes	Yes	Yes	Yes	Yes	Yes	No	Yes	Yes	8	Low
Pande et al.	Yes	Yes	Yes	Yes	Yes	Yes	No	Yes	No	7	Moderate
Patel et al.	Yes	Yes	Yes	Yes	Yes	Yes	Yes	No	No	7	Moderate
Qaserah et al.	Yes	Yes	Yes	Yes	Yes	Yes	Yes	No	Yes	8	Low
Roobthaisong et al.	Yes	No	Yes	Yes	Yes	No	No	Yes	Yes	7	Moderate
Roy et al.	Yes	Yes	Yes	Yes	Yes	Yes	Yes	Yes	Yes	9	Low
Sabir et al.	Yes	No	Yes	No	Yes	Yes	No	Yes	No	6	Moderate
Shah et al.	Yes	Yes	Yes	Yes	Yes	Yes	Yes	Yes	No	9	Low
Sinyange et al.	Yes	No	Yes	Yes	Yes	Yes	Yes	Yes	No	8	Low
Sule et al.	Yes	Yes	Yes	Yes	Yes	No	Yes	No	No	6	Moderate
Taher et al.	Yes	Yes	Yes	Yes	Yes	No	Yes	No	No	6	Moderate
Ujjiga et al.	Yes	No	Yes	Yes	Yes	Yes	No	Yes	Yes	8	Low
Wang et al	No	No	No	No	Yes	No	Yes	No	No	3	High
Winstead et al.	No	No	No	No	No	No	Yes	No	No	2	High
Zahid et al.	Yes	No	Yes	No	Yes	No	No	Yes	Yes	6	Moderate
Zgheir et al	Yes	Yes	Yes	Yes	Yes	Yes	Yes	Yes	No	8	Low
Zhao et al.	Yes	No	Yes	Yes	Yes	Yes	Yes	Yes	No	8	Low

References

1. Downes, M.J.; Brennan, M.L.; Williams, H.C.; Dean, R.S. Development of a Critical Appraisal Tool to Assess the Quality of Cross-Sectional Studies (AXIS). *BMJ Open* **2016**, *6*, e011458, doi:10.1136/bmjopen-2016-011458.
2. Muzembo, B.A.; Kitahara, K.; Debnath, A.; Ohno, A.; Okamoto, K.; Miyoshi, S.-I. Cholera Outbreaks in India, 2011-2020: A Systematic Review. *Int J Environ Res Public Health* **2022**, *19*, 5738, doi:10.3390/ijerph19095738.
