# Supplementary figures and images for: Cholera Outbreaks in Low- and Middle-Income Countries in the Last Decade: A Systematic Review and Meta-Analysis
Source: Microorganisms. 2024 Dec 4;12(12):2504. doi: 10.3390/microorganisms12122504 (PMC11728267; doi:10.3390/microorganisms12122504)

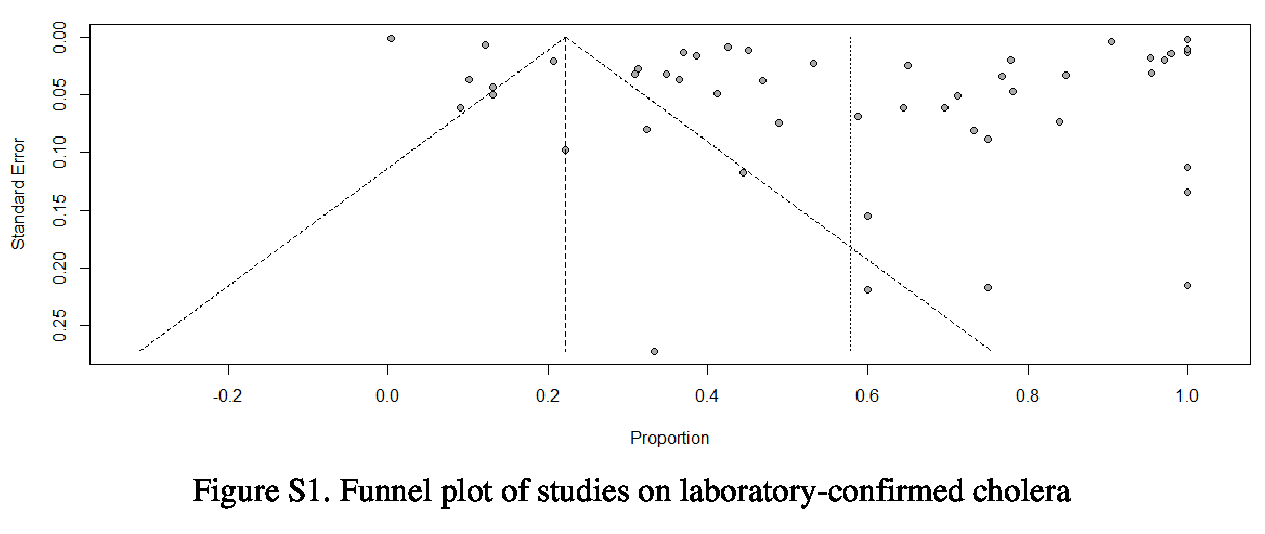

Supplement: Supplementary file 1 [file microorganisms-12-02504-s001.zip › Figure_S1.tiff]
